# Supplementary material for: A streamlined, nanopore-compatible 5PSeq protocol for rapid phenotypic antimicrobial sensitivity testing
Source: Cell Rep Methods. 2026 Mar 12;6(3):101327. doi: 10.1016/j.crmeth.2026.101327 (PMC13030957; doi:10.1016/j.crmeth.2026.101327)
Supplement: Document S1. Figures S1–S4 and Methods S1 [file mmc1.pdf]

Cell Reports Methods, Volume *6*

Supplemental information

A streamlined, nanopore-compatible 5PSeq protocol for rapid phenotypic antimicrobial sensitivity testing

Honglian Liu, Susanne Huch, Ryan Hull, Fabricio Romero Garcia, Lilit Nersisyan, Xiushan Yin, Wei-Hua Chen, Juan Du, and Vicent Pelechano

Supplemental Figures

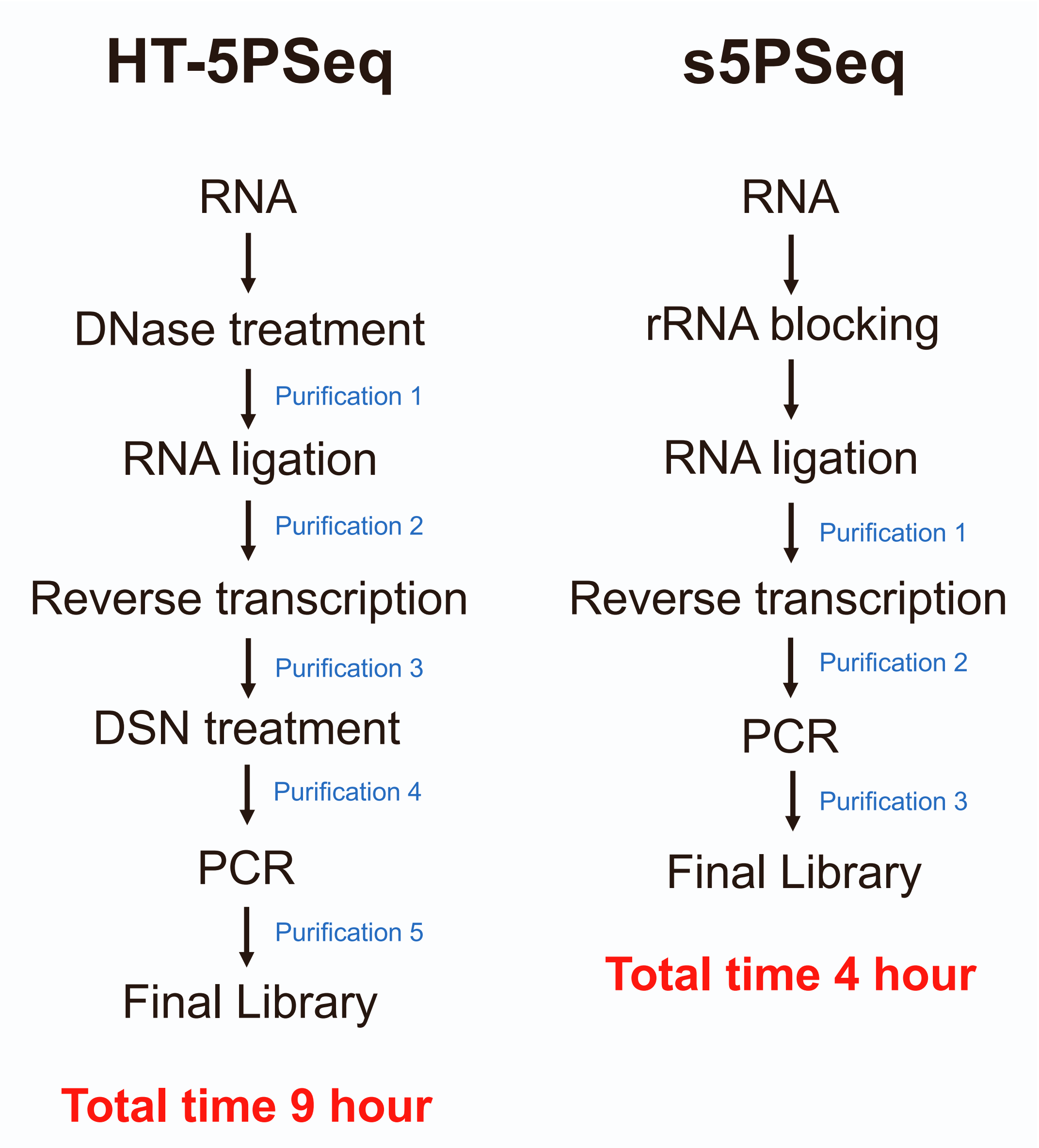

Figure S1: Comparison between HT-5PSeq and s5PSeq protocol, related to Figure 2.
The simplified s5PSeq method streamlines library preparation by reducing pipetting steps and minimizing purification, shortening the total workflow from 9 hours to 4 hours.

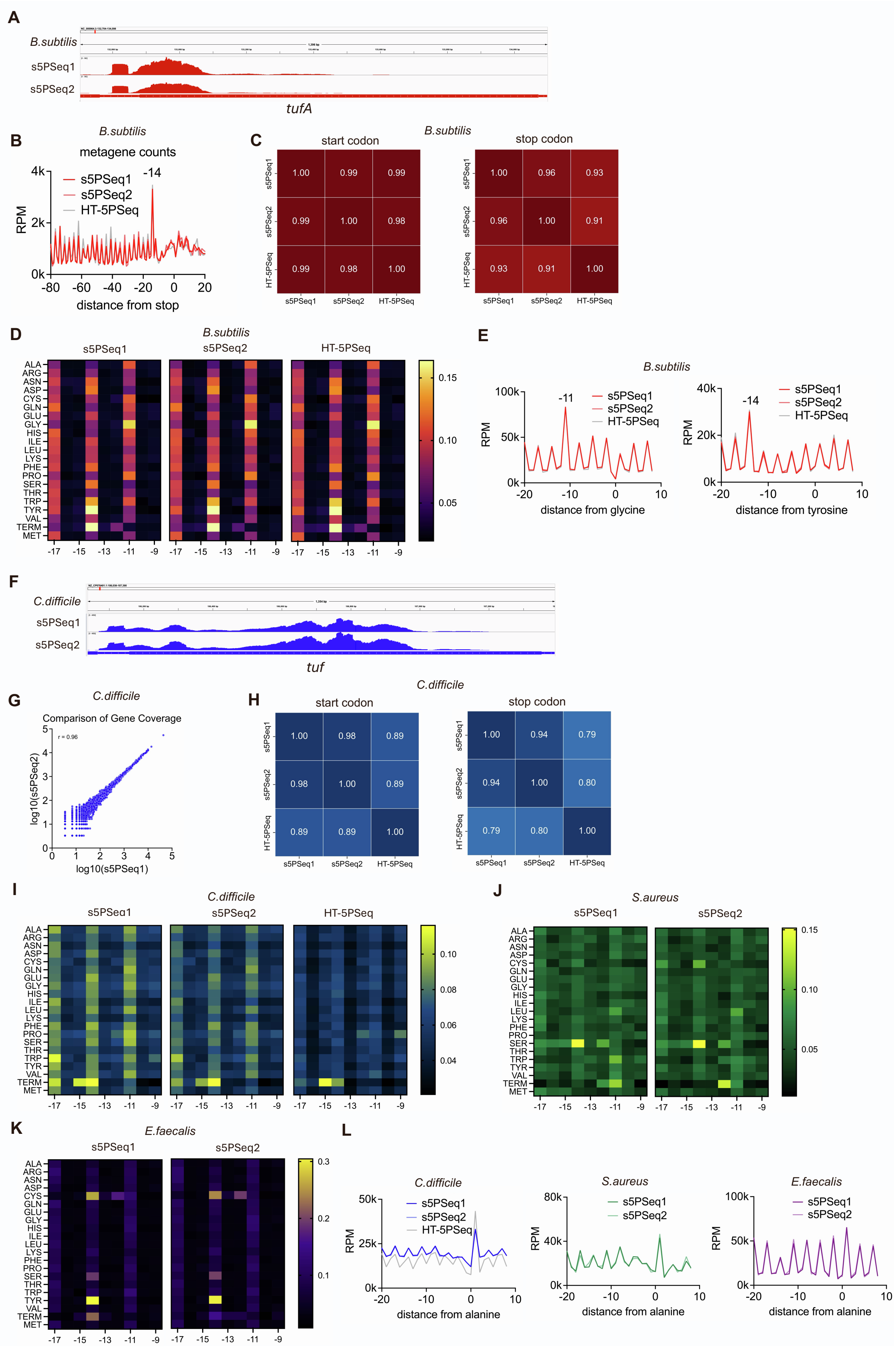
A
B.subtilis
s5PSeq1
s5PSeq2
tufA
B
B.subtilis
metagene counts
s5PSeq1
s5PSeq2
HT-5PSeq
-14
RPM
distance from stop
C
B.subtilis
start codon
stop codon
D
B.subtilis
s5PSeq1
s5PSeq2
HT-5PSeq
E
B.subtilis
distance from glycine
distance from tyrosine
F
C.difficile
s5PSeq1
s5PSeq2
tuf
G
C.difficile
Comparison of Gene Coverage
r = 0.96
log10(s5PSeq2)
log10(s5PSeq1)
H
C.difficile
start codon
stop codon
I
C.difficile
J
S.aureus
K
E.faecalis
L
C.difficile
S.aureus
E.faecalis
distance from alanine

Figure S2: Additional quality controls for s5Pseq method, related to Figure 2.

(A) Representative IGV coverage tracks for the highly expressed *tufA* gene in *B. subtilis*, showing highly consistent 5′P profiles between s5PSeq replicates (s5PSeq1 and s5PSeq2).

(B) Metagene analysis of 5'P mRNA read coverage relative to the stop codons in *B. subtilis*.

(C) Heatmap of Pearson correlation for normalized 5′P metagene coverage (RPM) around start codon (−50 to +50 bp) and stop codons (−80 to +20 bp) in *B. subtilis,* showing strong agreement between HT-5PSeq and s5PSeq replicates (s5PSeq1 and s5PSeq2).

(D) Heatmap showing amino acid-specific 5′P coverage obtained using s5Pseq (s5PSeq1 and s5PSeq2) and HT-5Pseq in *B. subtilis*. Positions at 14 and 11 nucleotides upstream of the amino acid codons correspond to ribosome occupancy at the A and P sites, respectively. For each amino acid codons, reads were normalized to the total number of 5′P reads within the −20 to −1 nucleotide window.

(E) Line plot showing 5'P mRNA degradation profiles at amino acid codon specific positions (x axis showing relative distance to glycine and tyrosine codons respectively) obtained using s5Pseq (s5PSeq1 and s5PSeq2) and HT-5Pseq in *B. subtilis.*

(F) Representative s5PSeq coverage tracks for the highly expressed *tuf* gene in *C.difficile*.

(G) Gene coverage comparison between replicates s5PSeq1 and s5PSeq2 in *C.difficile*, showing a strong linear correlation (Pearson's $r = 0.96$). Gene counts were normalized using median ratio normalization, followed by $\log_{10}$ transformation for visualization.

(H) Heatmap of Pearson correlation for normalized 5′P metagene coverage (RPM) around start codon (−50 to +50 bp) and stop codons (−80 to +20 bp) in *C.difficile* as in (C), showing strong agreement between HT-5PSeq and s5PSeq replicates (s5PSeq1 and s5PSeq2).

(I) Heatmap showing amino acid-specific 5′P coverage obtained using s5Pseq (s5PSeq1 and s5PSeq2) and HT-5Pseq in *C.difficile*, as in (D).

(J) Heatmap showing amino acid-specific 5′P coverage obtained using s5Pseq (s5PSeq1 and s5PSeq2) in *S.aureus*, as in (D).

(K) Heatmap showing amino acid-specific 5′P coverage obtained using s5Pseq (s5PSeq1 and s5PSeq2) in *E.faecalis*, as in (D).

(L) Line plot showing 5'P mRNA degradation profiles at amino acid codon specific positions (x axis showing relative distance to alanine codons) in *C.difficile*, *S.aureus* and *E.faecalis,* as in (E).

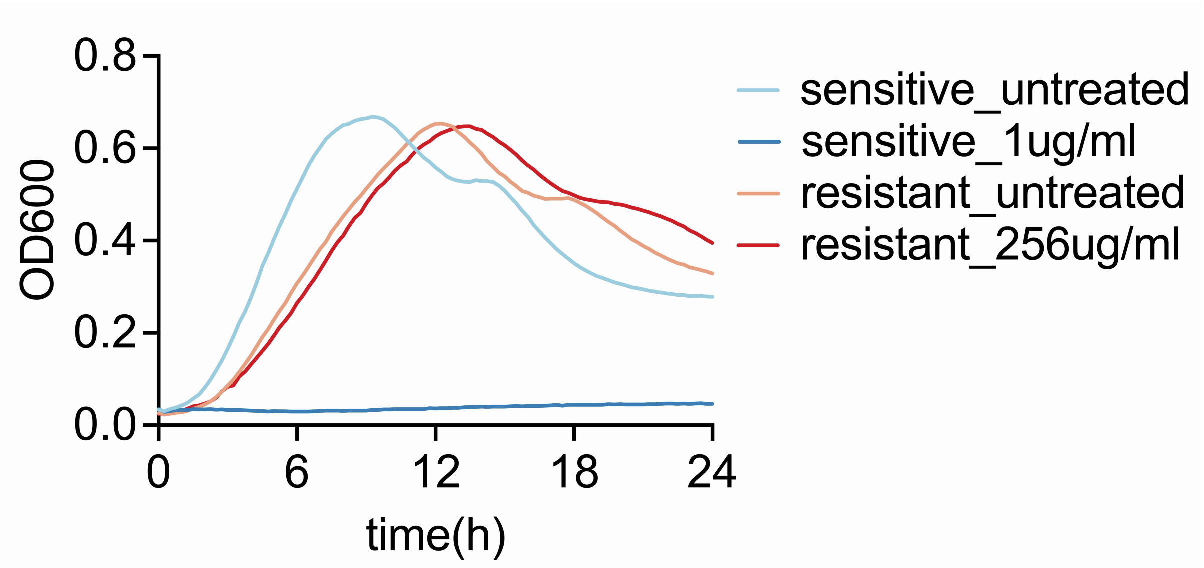

Figure S3: Growth curve of *C.difficile* clinical isolates, related to Figure 3. Erythromycin-resistant and –sensitive strains were grown in presence (treated) and absence (untreated) erythromycin at concentrations of 1 μg/mL and 256 μg/mL. Growth of the erythromycin-sensitive *C.difficile* strain is completely suppressed at 1 μg/mL, whereas the resistant strain exhibits robust growth at 256 μg/mL erythromycin concentration.

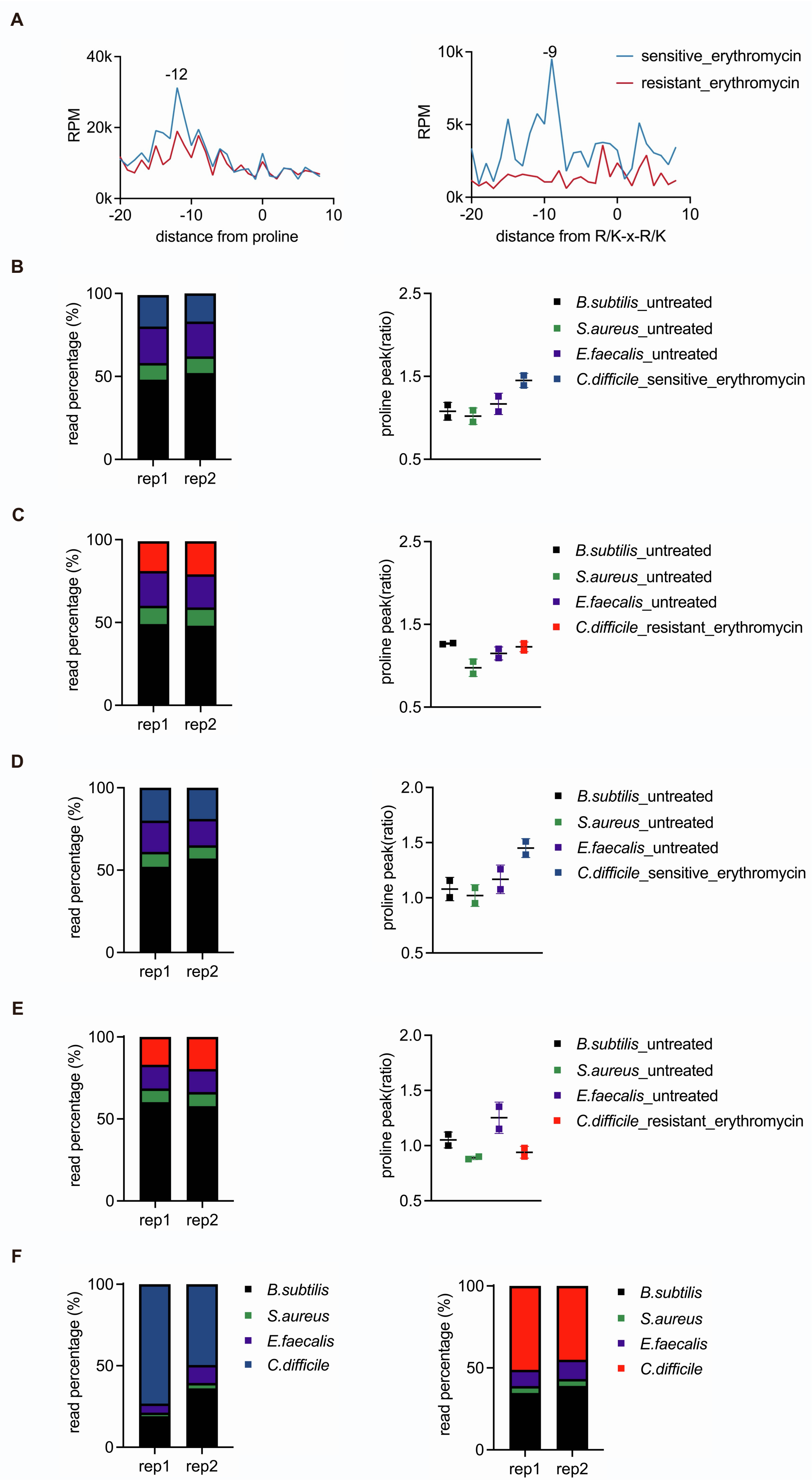

A
40k
20k
0k
RPM
-12
-20
-10
0
10
distance from proline
10k
5k
0k
RPM
-9
distance from R/K-x-R/K
sensitive_erythromycin
resistant_erythromycin
B
read percentage (%)
100
50
0
rep1
rep2
proline peak(ratio)
2.5
1.5
0.5
B.subtilis_untreated
S.aureus_untreated
E.faecalis_untreated
C.difficile_sensitive_erythromycin
C
C.difficile_resistant_erythromycin
D
2.0
1.5
1.0
0.5
E
F
B.subtilis
S.aureus
E.faecalis
C.difficile

Figure S4: Robust detection of erythromycin-induced ribosome stalling in *C. difficile* across sequencing platforms and mixed-species RNA backgrounds, related to Figure 4.

(A) Line plots showing erythromycin-induced ribosomal stalling in *C. difficile*, with clear differentiation between erythromycin-sensitive and -resistant strains, when 30,000 reads are subsampled from PromethION s5PSeq data shown in Fig. 4a. Stalling occurs at positions 12 nucleotides upstream of proline residues and 9 nucleotides upstream of the R/K-x-R/K motif (with the first base of the final codon in the motif set at position 0).

(B–C) Illumina NextSeq 2000 sequencing of mixed-species samples containing untreated RNA from *B. subtilis*, *S. aureus*, and *E. faecalis*, combined with erythromycin-treated *C. difficile*. Left bar plot shows species-level assignment of protein-coding reads for mixtures containing erythromycin-sensitive (B) or resistant (C) *C. difficile*, each with two biological replicates. Right dot plot shows corresponding peak-ratio metric quantifying context-specific ribosome stalling at positions 12 nt upstream of proline codons. Bars represent mean ± SD from n = 2 replicates. Strong erythromycin-dependent stalling is observed exclusively in mixtures containing the sensitive *C. difficile* strain, while no such stalling signature is detected in mixtures containing the resistant strain.

(D–E) Equivalent mixed-species experiments sequenced using a Nanopore MinION flow cell as in (B–C). Species composition and peak-ratio metrics recapitulate Illumina results, demonstrating platform-independent detection of erythromycin-induced stalling.

(F) Proportion of rRNA-derived Nanopore reads mapping to each bacterial species in the mixed-species samples as in (D–E). Reads were mapped to a combined multi-species reference using Minimap2, and only primary alignments with MAPQ ≥10 were retained. Species identities were inferred from rRNA annotations, enabling robust recovery of the expected species composition.

Methods S1: Detailed s5PSeq protocol, related to Figure 2

Starting Material: 200ng total bacterial RNA

1. **rRNA Blocking Oligo Hybridization (30min)**

In a 200 µL PCR tube, add the following reagents. Mix by gently flicking the tube and briefly spin down. Run the following program on a thermal cycler: 75 °C for 5 min; 65 °C for 5 min, and stepwise decrease of 3 °C every 2 min to 35 °C.

Reagent	Volume(µL)
Bacterial RNA (200ng)	1
Blocking oligo_16s5p (20µM) *	1
Blocking oligo_23s5p (20µM) *	1
Blocking oligo_universal (2µM) **	1
NaCl solution(250mM)	1
Total	5

*Species-specific oligos (see Table S2).
**Mixture of 146 oligos (see Table S2).

2. **ssRNA Ligation (1hour)**

To the hybridized sample, add the following reagents. Mix by flicking the tube and briefly spin down. Incubate at 25 °C for 1 hour with the lid temperature off.

Reagent	Volume(µL)
rP5_RND adapter (10µM)	1
10X T4 RNA ligation buffer	1
T4 RNA ligase 1 (10U/µL) ***	1
Nuclease-free H2O	1
ATP (10mM)	1
Sample from Step 1	5
Total	10

*** T4 RNA ligase 1 can be substituted with other single-stranded RNA ligases with comparable 5′-phosphate RNA ligation activity.
Note: PEG 8000 is omitted during the ssRNA ligation step. Although PEG is typically used to enhance ligation efficiency, we found that excluding it improves mRNA recovery, likely by reducing the efficiency of unintended ssRNA ligation to abundant rRNA species.

3. **Bead Purification (6min)**

1) Add 30 µL nuclease-free H_2O to the 10 µL sample (final volume: 40 µL).
2) Add 72 µL (1.8X) RNA CleanXP beads (Beckman Coulter) and mix thoroughly by pipetting.
3) Incubate at room temperature for 3 min.
4) Place the tube on a magnetic stand until the solution clears.
5) Discard the supernatant. Wash the beads 3 times with 200 µL freshly prepared 70% ethanol.
6) Air-dry the beads, then elute RNA in 10 µL nuclease-free H_2O.
7) Incubate at room temperature for 3 min, place on magnet, and transfer the supernatant to a new tube.

Note: RNA CleanXP beads can be substituted with other SPRI-based magnetic beads exhibiting comparable RNA-binding properties, including homemade preparations. Philippe Jolivet, Joseph W. Foley 2020. SPRI bead mix. **protocols.io** https://dx.doi.org/10.17504/protocols.io.bnz4mf8w

4. **Reverse Transcription (1hour 20min)**

1) Add the following to the purified RNA, incubate at **65 °C for 5 min**, then place immediately on ice.

Reagent	Volume(uL)
Random hexamer (20μM)	1
dNTPs (10mM)	1
Sample from Step 3	10
total	12

2) Add the following reagents, then run this program: 25 °C, 10 min; 42 °C, 50 min; 70 °C, 15 min.

Reagent	Volume(uL)
5X First-strand buffer	4
DTT (0.1M)	2
Nuclease-free H2O	1
SuperScript II (200U/μL) ****	1

****SuperScript II (200U/μL) can be substituted with other reverse transcriptase with equivalent activity such as RT-MashUP (Alekseenko et al. Sci Rep. 2021doi: 10.1038/s41598-020-80352-8)

5. RNA Depletion and Beads Purification (30 min)

1) Add 8 μL 100 mM NaOH to each sample and incubate at 65 °C for 20 min to hydrolyze RNA.
2) Neutralize with 8 μL 100 mM Tris-HCl.
3) Add 65 μL (1.8X) AMPure XP beads (Beckman Coulter), mix well, and incubate at room temperature for 3 min.
4) Place the tube on a magnetic stand, wait until clear, discard supernatant.
5) Wash beads 3 times with 200 μL freshly prepared 70% ethanol.
6) Air-dry beads and elute DNA in 10 μL nuclease-free H_2O.
7) Incubate at room temperature for 3 min, place on magnet, and transfer the supernatant to a new tube.

Note: AMPure XP beads can be substituted with other SPRI-based magnetic beads with comparable DNA-binding properties, including homemade alternatives.

6. PCR Amplification (30 min)

Add the following reagents and run the PCR program below.

Reagent	Volume(uL)
2X Phusion HSII High-Fidelity master mix*****	9
PE1 (NEBi5, 10μM)	0.5
PE2 (PE2_MPX, 10μM)	0.5
Sample from previous step	10
total	20

*****2X Phusion HSII High-Fidelity master mix can be substituted with other high-fidelity DNA polymerases with dNTP-containing buffer systems and comparable performance.

PCR Program:

Temperature	Time	Cycles
98ºC	30 sec	1
98ºC	20 sec	
65ºC	30 sec	15X
72ºC	30 sec	
72 ºC	7 min	1
4 ºC	Hold	∞

7. Library Purification and Size Selection (10 min)

1) Add 80 µL nuclease-free H_2O to bring total volume to 100 µL.
2) Add 60 µL (0.6X) AMPure XP beads, mix thoroughly, and incubate at room temperature for 3 min.
3) Place on magnet, transfer 160 µL supernatant to a new tube containing 20 µL (0.2X) AMPure XP beads, mix, and incubate 3 min.
4) Place on magnet, discard supernatant, and wash beads 3 times with 200 µL freshly prepared 70% ethanol.
5) Air-dry beads and elute DNA in 10 µL nuclease-free H_2O.
6) Incubate at room temperature for 3 min, place on magnet, and transfer 10 µL supernatant to a clean tube.
7) Quantify libraries using Qubit and assess size distribution with Bioanalyzer.

8. Sequencing Compatibility

- Illumina: Libraries are directly compatible with Illumina sequencing platforms.
- Nanopore: For Oxford Nanopore sequencing, libraries can be used as template for the the Direct Ligation Kit v14 (SQK-LSK114) following the manufacturer's protocol (~1 hour).

Table: Time and Estimated Per-Sample Cost of s5PSeq Library Preparation

Step	Time (hour)	Cost (USD)
1. rRNA Blocking Oligo Hybridization	0.5	0.1
2. ssRNA Ligation	1.0	0.7
3. Bead Purification	0.1	1.5
4. Reverse Transcription	1.3	4.0
5. RNA Depletion and Beads Purification	0.4	1.3
6. PCR Amplification	0.5	0.9
7. Library Purification and Size Selection	0.2	1.5
**Total**	~4	~10

Note: Total costs are estimated based on commercial reagent pricing at small-scale use (per sample basis). Significant reductions are possible using homemade SPRI bead protocols and open-source enzyme preparations (~2 USD per sample), with comparable performance (data not shown).
