# Supplementary material for: A streamlined, nanopore-compatible 5PSeq protocol for rapid phenotypic antimicrobial sensitivity testing
Source: Cell Rep Methods. 2026 Mar 12;6(3):101327. doi: 10.1016/j.crmeth.2026.101327 (PMC13030957; doi:10.1016/j.crmeth.2026.101327)
Supplement: Data S1. Interactive FivePSeq html report, related to Figure 2 [file mmc4.zip › bsub_s5pseq_to_compress/fivepseq_plots/comparison/bsub_differential_heatmaps.html]

bam\_differential\_heatmaps
